# Prognosis of Guillain-Barré Syndrome in Children

**Published:** 2016

**Authors:** Mohammad Reza SALEHIOMRAN, Ali NIKKHAH, Mohadese MAHDAVI

**Affiliations:** 1 Non-Communicable Pediatric Diseases Research Center, Amirkola Children’s Hospital, Babol University of Medical Sciences, Babol, Iran; 2 Pediatric Neurology Department, Amirkola Children’s Hospital, Babol University of Medical Sciences, Babol, Iran; 3 Pediatric Department, Amirkola Children’s Hospital, Babol University of Medical Sciences, Babol, Iran.

**Keywords:** Prognosis, Guillain-Barre, Children

## Abstract

**Objective:**

Guillain-Barre Syndrome (GBS) is an acute polyradiculoneuropathy characterized by progressive motor weakness of limbs and areflexia. In this study, our aim was to evaluate the clinical pattern and prognosis of children with Guillain-Barre syndrome.

**Materials & Methods:**

This cross-sectional study was conducted in the Pediatric Neurology Unit of Amirkola Children’s Hospital, Babol, Iran during the period of 5 years from October 2008 to September 2013. We assessed the clinical features, results of electrodiagnostic tests, functional status, treatment and outcome of 17 children diagnosed with GBS.

**Results:**

Of 17 (male to female ratio = 1.6:1) children studied, all had motor weakness, 4 children (23.5 %) and cranial nerve palsies. Respiratory paralysis was found in one child requiring assisted ventilation. Antecedent illness preceding GBS was recorded in 7 (41.2%) children. The GBS subtype distribution as per electrodiagnostic studies was as follows: acute inflammatory demyelinating polyradiculoneuropathy (AIDP) in 12 (70.6%) acute motor axonal neuropathy (AMAN) in 3 (17.6%), acute motor sensory axonal neuropathy (AMSAN) in 2 (11.8%). IVIG constituted the treatment given in all of the patients. Complete recovery was observed in 16 children and the remaining one child was dependent to wheelchair.

**Conclusion:**

GBS in children is not poor prognostic disorder and our recommendation is administration of IVIG as soon as possible after clinical diagnosis. Except for one child who remained wheelchair bound, there was no mortality or morbidity in long-term observation. Besides, strong limitation of our study was the low number of subjects.

## Introduction

Guillain-Barré syndrome (GBS) is an immune mediated polyradiculoneuropathy with an acute onset and a variable clinical course (1). It is also known as Landry’s paralysis and frequently preceded by an unspecified infection (2). This syndrome manifests as progressive areflexic or hyporeflexive motor paralysis with or without sensory deficit. Weakness typically progress over hours to a few days (2). Autonomic dysfunction is common in GBS. Recovery period in this disorder is shorter in children than adults are and mortality rate in children is about 3-5% (1). Respiratory insufficiency is an ominous event in GBS and can lead to death in these patients. 

Besides, autonomic dysfunction is another main cause of mortality in affected children (1,3). Nerve conduction velocity (NCV) is performed to confirm this diagnosis.

IVIG and plasma exchange have made a significant change in the course of the illness (4).

We performed a cross sectional study on the natural history in children with GBS to study their clinical profile using intravenous immunoglobulin (IVIG) in addition to supportive management in long-term assessment (from2008 to 2014).

## Materials & Methods

This cross-sectional study was conducted in the Pediatric Neurology Unit of Amirkola Children’s Hospital, Babol Medical University, Babol, Iran during the period of 5 years from October 2008 to September 2013. The subjects of the study were children < 10 yr of age diagnosed with GBS. We assessed the clinical features, results of electro-diagnostic tests, functional status, treatment instituted and outcome.

A complete neurological examination was conducted including examination of cranial nerves, motor system and sensory system as well as deep tendon reflexes. Electrodiagnostic study was done in all patients and electrophysiological subtypes of GBS were noted. CSF examination was performed in selected hemodynamically stable children.

All children were treated with IVIG in a dose of 2 gm/kg over 2-5 d in addition to conventional supportive and/or intensive respiratory care. 

Statistical analysis was done using SPSS (Chicago, IL, USA) and numerical parametric data were presented as percentages.

This study has been done with approval of the Ethics Committee of the Babol University of Medical Sciences. 

All patients’ children were taken informed consent form.

## Results

We evaluated 17 children with documented GBS admitted in our hospital and followed them four times (3, 6, 12, 24 months) after discharge. Eleven (64.7%) were male. Age of our patients on admission time ranged from 2 yr to 9 yr (4.4± 1.9). The history of antecedent illness was found in 7 subjects (41.2%) in the preceding two weeks. Of these antecedent illnesses, respiratory tract infection was found in 3 cases (42.9%), diarrhea in 2 cases (28.6%) and nonspecific febrile illness in 2 children (28.6%).

At admission, all 17 children presented with limb weakness. Cranial nerve palsy was observed in 4 (23.5%) children. Of which, 2 had bulbar palsy and 2 children had facial palsy. Respiratory insufficiency was found in one child requiring assisted ventilation. The GBS subtype distribution as per electrodiagnostic studies was as follows: acute inflammatory demyelinating polyradiculoneuropathy (AIDP) in 12 (70.6%), acute motor axonal neuropathy (AMAN) in 3 (17.6%) and acute motor sensory axonal neuropathy (AMSAN) in 2 (11.8%). NCV (nerve conduction velocity) was performed in all children at first 72 h of admission time except in one child intubated and NCV was done after extubation. CSF analysis was performed in 11 children at 5-7th days of admission and the characteristic feature of albumin-cytological dissociation (<10 mononuclear cells) was observed in only two children. IVIG was administered to all patients (2 gm/kg) in five consecutive days. Physical therapy was ordered for all patients.

We followed all patients for long time. In first visit after about three months, 6 (35.3%) patients had normal neurologic examination. In second visit after about six months, 12 (70.6%) patients were normal. in third visit after about 12 months after discharging from the hospital, 16 (94.1%) patients were normal and had complete motor function with normal deep tendon reflexes and only one child was wheelchair bound and finally in last visit after 24 months (about 2 yr), our findings was similar to third visit.

## Discussion

GBS is the most common cause of acute flaccid paralysis in infants and children. GBS could be seen in all age groups. We included, 17 cases of <10 yr of age (range 2-9) and 41% of children were below 4 yr. In one study of 61 children younger than 15 yr of age with GBS, found that most of the children who had GBS were below 4 yr, and only one case was seen in the 10-15 yr age group (4). 

In our study, male: female ratio was 1.8:1. Dhadke et al., observed a male preponderance in their study with a male: female ratio of 1.5:1 (2). Maneesh Kumar et al., reported a male: female ratio of 2.3:1 (8).

Two third of the cases will develop the neurological signs and symptoms within one to two weeks from the antecedent illness (8). In this study, history of antecedent illness was found in 7 subjects (41.2%) in the preceding two weeks. Dhadke et al., reported respiratory infections as the most common preceding disorder in their study, followed by gastrointestinal infection (2). All subjects in our study presented with limb weakness. Akbayram et al., reported limb weakness in 34/36(94.4%) of children with GBS (6). Cranial nerve palsy was observed in 4 (23.5%) children. Loeffel et al., reported a 50% incidence of cranial nerve palsies in GBS, facial nerve being the commonest (7). Respiratory insufficiency was found in one child requiring assisted ventilation. Akbayram et al, reported respiratory insufficiency and assisted ventilation in 3/36 (8.3%) of children with GBS (6). In our study, the most common form of electrophysiologic form of GBS was AIDP (70.6%). Most of the pediatric patients in the study by Pi-Lien et al., belonged to the AIDP group (3). In Akbayram et al study, AIDP was the most common form (69.4%) (6). CSF analysis was performed in 11 children. Typical CSF finding of less than 10 mononuclear leukocytes/mm3 was found in only 2 cases. Albumino-cytological dissociation in CSF analysis (elevated CSF protein with less than 10 mononuclear WBCs / mm3) was reported in 2 out of 5 children in a recent study (8).

Fortunately, we did not see any death due to GBS in our long-term follow up study, while Akbayram et al, reported 8.3% deaths (3/36) (6). Our study was similar to Maneesh Kumar et al. study (8). (Table 1).

Finally, complete recovery after last visit (after 24 months) was 16/17 (94.1%) in our study. In Korinthenberg et al study, complete recovery was 92% and in Maneesh Kumar et al. study was 82.4% (8, 9).

**In conclusion, **GBS is not poor prognostic disorder in children, especially; we administered IVIG as soon as possible after clinical suspicion. Regardless of the severity of disease on admission time and electrophysiological subgroups, a majority achieved complete motor and sensory recovery. Strong limitation of our study was the low number of cases. Except for one child who remained wheelchair bound, there was no mortality or morbidity in long term observation. 

**Table 1:** Comparison of Prognosis of Subjects with Other Two Studies in Terms of Number (Percent

**Prognosis (%)**	**Akbayram S et al(6)**	**Maneesh Kumar et al(8)**	**Our study**
**Complete Recovery**	*29(80.5%)*	14(82.4%)	16(94.1%)
**Incomplete Recovery**	*4(11.2%)*	3(17.6%)	1(5.9%)
**Death**	*3(8.3%)*	-	-
**Total subjects **	36	17	17
